# Dissecting the Interaction Deficiency of a Cartilaginous Fish Digestive Lipase with Pancreatic Colipase: Biochemical and Structural Insights

**DOI:** 10.1155/2020/3064290

**Published:** 2020-03-13

**Authors:** Neila Achouri, Màrius Tomàs-Gamisans, Soumaya Triki, Francisco Valero, Nabil Miled, Ahmed Fendri, Nabil Smichi

**Affiliations:** ^1^University of Sfax, ENIS, Laboratory of Biochemistry and Enzymatic Engineering of Lipases, Road of Soukra, BPW 1173-3038 Sfax, Tunisia; ^2^UAB, Universitat Autònoma de Barcelona, Departament d'Enginyeria Química, Biològica i Ambiental, Bellaterra Barcelona, Spain; ^3^University of Sfax, Center of Biotechnology of Sfax, Laboratory of Molecular and Cellular Screening Processes, BP 111 7 Road Sidi Mansour km 6, Sfax, Tunisia; ^4^University of Jeddah, College of Science, Department of Biological Sciences, Jeddah, Saudi Arabia; ^5^Functional Genomics and Plant Physiology Unit, Higher Institute of Biotechnology of Sfax, 3038 Sfax, Tunisia; ^6^Mayo Clinic Arizona, 13400 Shea Boulevard, Scottsdale, AZ 85259, USA

## Abstract

A full-length cDNA encoding digestive lipase (SmDL) was cloned from the pancreas of the smooth-hound (*Mustelus mustelus*). The obtained cDNA was 1350 bp long encoding 451 amino acids. The deduced amino acid sequence has high similarity with known pancreatic lipases. Catalytic triad and disulphide bond positions are also conserved. According to the established phylogeny, the SmDL was grouped with those of tuna and *Sparidae* lipases into one fish digestive lipase cluster. The recently purified enzyme shows no dependence for bile salts and colipase. For this, the residue-level interactions between lipase-colipase are yet to be clearly understood. The structural model of the SmDL was built, and several dissimilarities were noticed when analyzing the SmDL amino acids corresponding to those involved in HPL binding to colipase. Interestingly, the C-terminal domain of SmDL which holds the colipase shows a significant role for colipase interaction. This is apt to prevent the interaction between fish lipase and the pancreatic colipase which and can provide more explanation on the fact that the classical colipase is unable to activate the SmDL.

## 1. Introduction

Pancreatic lipases (triacylglycerol hydrolase, EC 3.1.1.3) are carboxylester hydrolases that catalyse the hydrolysis of fats and oils into glycerol and free fatty acids at the oil/water interfaces [1–4]. They are much more active on insoluble substrates than on soluble esters; this property has been designated as “interfacial activation” [5]. In the presence of bile salts, mammalian pancreatic lipase activity requires a small protein coenzyme called colipase that allows the enzyme to bind to the triacylglycerol/water interface. The amino acid sequence of the porcine pancreatic lipase was first published in 1981 [6], and since then, several mammalian pancreatic lipases have been isolated and characterized [7–10]. Thanks to the resolution of numerous three-dimensional structures, a better comprehension has been reached for the pancreatic lipase structure-function relationships [10–12]. Van Tilbeurgh et al.were the first to determine the 3-D structure of HPL, followed directly by the 3-D structure of a HPL-procolipase complex in the presence or in the absence of mixed phospholipid/bile salt micelles, as well as in complex with a C_11_ alkyl phosphonate inhibitor [12]. The lipase is a single polypeptide chain of 449 amino acids and is folded into two structural domains [10]: a large N-terminal domain (residues 1-336) and a smaller C-terminal domain (residues 337-449). The N-terminal domain belongs to the *α*/*β*-hydrolase fold [13] and contains a catalytic triad formed by serine, aspartic acid, and histidine that is analogous to that of serine proteases [14, 15]. While the N-terminal domain carries the catalytic activity, the *β*-sandwich C-terminal domain plays an important structural role as it binds specifically to the colipase. This cofactor exposes the hydrophobic tips of its fingers at the opposite side of its lipase-binding site through noncovalent interactions [10, 13]. In recent years, researchers have been seeking to widen the study of pancreatic lipases from birds [8, 16] and invertebrates [14, 17, 18]. The turkey pancreatic lipase (TPL) shows the same features of HPL, and its three-dimensional structure consists of two domains stabilized by six disulphide bridges [8]. Moreover, other studies have reported new enzymes isolated from aquatic species with interesting potential in food processing [3, 19–21]. To provide an overview on pancreatic lipases, we have tried to extend our research on the fish lipolytic system in order to understand the evolutionary aspects of fish lipases as compared to those of mammals. Previously, many studies have been carried out on digestive lipases of bony fish from the grass carp [22] and Pengze crucian carp [23]. The *Sparidae* family has been considered with regard to lipolytic enzymes involved in lipid digestion; Smichi et al. [24] have purified a lipase from the annular seabream (AsDL). The characterized enzyme shows distinct properties to TPL [8] and lipases from other marine species [19, 21]. From the same family, a lipase from the red seabream has been isolated and purified and its functional properties have been performed to explain the differences with pancreatic lipases [25]. Yet, little information is available regarding structure-function relationships of cartilaginous fish (chondrichthyans) lipases.

The common smooth-hound (*Mustelus mustelus*) as one of the chondrichthyans and the most used for human consumption in Tunisia [26] was used as a model in this study. This species possesses a complex digestive system with a pancreas secreting digestive enzymes. Recently, we have purified and characterized the SmDL from the delipidated pancreatic powder of smooth-hound [27]. The SmDL molecular weight was around 50 kDa. The purified enzyme hydrolyses efficiently triacylglycerols with 2200 U/mg on tributyrin as a substrate and was found to be stable at 50°C. Unlike known mammal pancreatic lipases, this enzyme was not activated by the pancreatic colipase. There is a little information about pancreatic lipases from chondrichthyans. Here, we report for the first time the isolation and the cloning of the gene coding for the SmDL. For the sake of comparison, the gene coding the mature lipase from the European eel *A. anguilla* (EeDL) was cloned and sequenced. A phylogenetic tree has been generated in order to determine the evolutionary line of chondrichthyan lipases with other digestive lipases. Using the three-dimensional structure of the HPL as a template, structural models of the SmDL and EeDL were built and used to provide explanation on the poor interaction of the SmDL with the classical colipase.

## 2. Materials and Methods

### 2.1. Strains, Plasmids, and Chemicals

*E. coli* strain XL1 blue was used as the cloning host and was grown in Luria-Bertani (LB) medium [28], supplemented with 10 *μ*g/mL tetracycline and 100 *μ*g/mL ampicillin. A pGEM-T Easy Cloning Vector was from Promega (France). The PCR products were purified using a QIAquick PCR Purification Kit (Qiagen). IPTG and X-Gal (isopropyl-thio-*β*-d-galactopyranoside and 5-bromo-4-chloro-3-indolyl-beta-D-galactopyranoside) were bought from Global Biotechnology. All oligonucleotides and enzymes used in DNA manipulations were from Invitrogen. THL (tetrahydrolipstatin) was a generous gift from Pr. A.F. FISCHLI (Buchs, Switzerland). NaDC (sodium deoxycholate) was from Sigma Chemical (St. Louis, USA).

### 2.2. Biological Materials

Fresh common smooth-hound *(M. mustelus*) and European eel (*A. anguilla*) were caught and transported forthright in cold ice (4°C) to the laboratory. Fresh pancreases from each species were dissected and immediately used for total RNA extractions.

### 2.3. Pancreatic Lipases

The pancreatic lipases SmDL and TPL were purified as previously described [16, 27].

### 2.4. Pancreatic Colipase Preparation

In order to check the presence of colipase in the common smooth-hound's pancreas, the delipidated powder homogenate was treated during 15 min at pH 2 and at 60°C to inactivate lipase and maintain only colipase activity. To study the lipase interaction with colipase, SmDL and TPL activities were measured using olive oil emulsion as a substrate in the presence and absence of colipase, using the pH stat method under optimal conditions of pH and temperature as described previously [16, 27].

### 2.5. RNA Extraction and Cloning of SmDL and EeDL

Total RNA was isolated from 0.1 g of both fish pancreases using a TRIzol reagent as described by the manufacturer's protocol (Invitrogen). First-strand cDNAs were synthesized in 20 *μ*L by reverse transcribing 2 *μ*g of total RNA, using 1 *μ*L of M-MLV as the reverse transcriptase (200 unites) and 10 *μ*M oligo (dT)_18_ as the primer according to the manufacturer's instructions. First-strand cDNAs were treated with 1 *μ*L RNase inhibitor (40 U/*μ*L), and the part of the cDNAs encoding for mature lipases was amplified using the oligonucleotides listed in Table 1. Primers were predicted from putative pancreatic lipases of elephant shark (*Callorhinchus milii*, GenBank accession number PRJNA18361) and Japanese eel (*Anguilla japonica*, GenBank accession number AB070722.1) for amplification of smooth-hound (SmDL) and European eel (EeDL) genes coding for digestive lipases, respectively. PCR amplifications were realized by Pfu DNA Polymerase (Promega) according to the enzyme instructions. The PCR mixture (20 *μ*L) contained 1 *μ*g of 1^st^-strand cDNA, 1.25 *μ*L of each specific oligonucleotide primer (25 *μ*M) (Table 1), 0.25 *μ*L of Pfu DNA Polymerase (2-3 U/*μ*L), and 0.2 mM of deoxynucleoside triphosphate. Amplification was realized in a Bio-Rad thermal cycler, utilizing the reaction settings as follows: initial denaturation at 95°C for 2 min, followed by 35 cycles of denaturing at 95°C for 1 min, annealing temperature for 30 s, extension at 72°C for 3 min, and a final extension at 72°C for 5 min. PCR fragments (approximately 1350 bp) were purified and ligated into the dephosphorylated pGEM-T Easy Vector according to the manufacturer's protocol. After transformation into the competent XL1 blue cells, the recombinant colonies were identified through blue-white color selection in IPTG, X-Gal, and ampicillin-containing LB plates and confirmed by PCR colony followed by sequencing on both strands using an ABI PRISM 3100 sequencer. The nucleotide sequences of the two mature lipases (SmDL and EeDL) determined in this study were deposited in the GenBank database under accession numbers KY548033 and KY548034, respectively.

### 2.6. Database Research and Phylogenetic Studies

The reading frame of the two sequenced cDNAs was translated using the ExPASy translate tool program, and the deduced fish digestive lipase amino acid sequences were compared using the BLASTP algorithm with sequences already existing in the NCBI Protein Database. Sixteen homologous digestive lipase sequences from different mammals, fish, and birds were used for multiple sequence alignment using BioEdit v.7.2.5 and the default settings [29]. The output of the BioEdit multiple sequence alignment was color coded according to their identity. The amino acid sequences of lipases from different species were aligned. Using the maximum parsimony method, the phylogenetic trees were carried out in Molecular Evolutionary Genetics Analysis (MEGA6) [30, 31]. The branch robustness was assessed by bootstrap analysis of 100 replicates of resampling, and only values that were highly significant (≥70) are shown [32]. All positions containing gaps and missing data were eliminated. There were a total of 442 positions in the final dataset. Evolutionary analyses were carried out in MEGA6 [31].

### 2.7. 3-D Structure Modeling

The 3-D structure models of SmDL and EeDL were built under an active (open) form using the 3-D structure of the active form of human pancreatic lipase (1LPB) as a template. The models were built by homology by submitting the alignment generated by BioEdit v.7.2.5 [29] to the automated structure-modeling SWISS-MODEL workspace [33]. In order to optimize the structures, the model was subjected to three cycles of minimization, each containing 50 steps of conjugate gradient, using the GROMOS96 software implemented to Swiss-PdbViewer [34]. The cutoff was set to 10 Ǻ and a harmonic constraint was used. The geometry quality of the final model was checked using the PROCHECK program [35]. The figure was generated using PyMOL (http://www.pymol.org).

## 3. Results and Discussion

### 3.1. Identification of SmDL and EeDL Lipase Coding Genes

**Figure 1:**
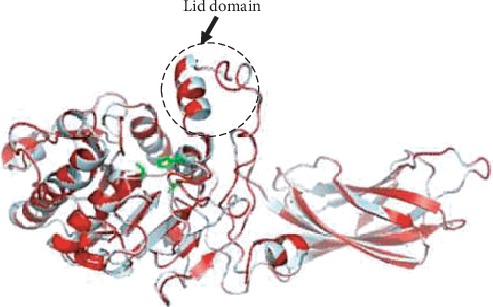
Cartoon presentation of structure models of SmDL in red superimposed with the human pancreatic lipase (in cyan). The catalytic triad residues are shown as green sticks, and the lid domain under its open form is indicated.

**Figure 2:**
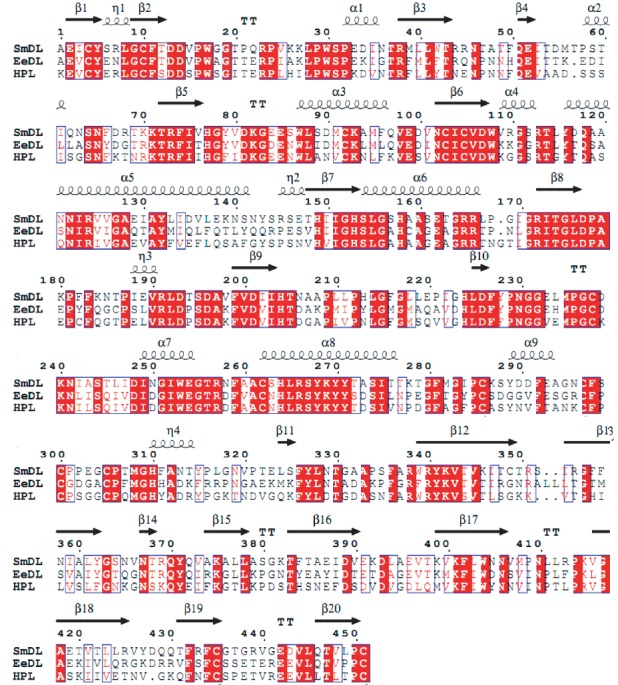
Alignment of the amino acid sequences of the mature forms of SmDL and EeDL with HPL. Boxes in light grey indicate positions at which the amino acids are identical in the three lipases. The closed arrows indicate the Ser, Asp, and His which form the catalytic triad. The dashes represent gaps introduced during the alignment process. The secondary structures of *Sparidae* lipases are indicated. Homology alignment was performed using the software BioEdit v.7.2.5.

Total RNAs were obtained from *M. mustelus* ([Supplementary-material supplementary-material-1], lane 1) and A. anguilla ([Supplementary-material supplementary-material-1], lane 2) pancreases. The analyzed products showed RNA high qualities that will be used as templates for gene amplification. Full-length coding sequences for the SmDL and EeDL were obtained from *M. mustelus* and *A. anguilla* products, respectively, by the reverse transcriptase-PCR technique and using the appropriate primers as described in Material and Methods. Amplified cDNA fragments (about 1350 bp) were electrophoretically separated ([Supplementary-material supplementary-material-1]). These cDNA products were ligated in a pGEM-T Easy Vector. The recombinant vector was transformed into *E. coli* cells. The white colonies (positives clones) were randomly selected, and the presence of SmDL and EeDL inserts in the plasmid was checked by colony PCR. Vectors having each insert were sequenced using T7 and SP6 primers. The sequences of all fragments were aligned by BioEdit v.7.2.5 [29]. The complete sequences of SmDL and EeDL ([Supplementary-material supplementary-material-1]) consisting of 1.35 kb were submitted in the GenBank and were assigned the accession numbers KY548033 and KY548034, respectively. The nucleotide BLAST analysis for SmDL showed that this enzyme shared high identity (77%–87%) with pancreatic lipase genes from other cartilaginous fish: 87% with the whale shark (*Rhincodon typus*), 82% with the common stingray (*Dasyatis pastinaca*) [36], and 77% with the elephant shark (*Callorhinchus milii*). The EeDL ([Supplementary-material supplementary-material-1]) shared high identities of 96% and 75% with the Japanese eel (*Anguilla japonica*) and hareng (*Clupea harengus*) lipase genes, respectively. The deduced polypeptide sequences of SmDL and European eel digestive lipases comprise 451 and 452 amino acids, respectively. High amino acid sequence identities were found between SmDL and other cartilaginous fish lipases like *Callorhinchus milii* (68%) and *Dasyatis pastinaca* (79%) [36]. The EeDL amino acid sequence displayed prominent similarity with those of other fish species like *Anguilla japonica* (97%) and red seabream (63%). SmDL and EeDL structures superposition with the human pancreatic lipase (HPL) is shown in Figure 1. Both enzymes possess two and three supplementary amino acids, respectively, as compared to the HPL (449 amino acids). Residues of the HPL catalytic triad (Ser153, Asp177, and His 264) are conserved in both fish lipases. The highest homology was observed around the active-site serine for the three lipases (Figure 2). A comparison of the N- and C-terminal domains of SmDL and EeDL with that of HPL shows that the N-terminal domain is more conserved (about 63%) than the C-terminal domain (about 47%). Sequences corresponding to the HPL lid domain and *β*5 loop [13] are conserved for the two fish lipases. The twelve cysteine residues involved in disulphide bridges in the case of HPL are also conserved for SmDL and EeDL, suggesting also the presence of 6 disulphide bridges in the two fish digestive lipase structures (Figure 2).

### 3.2. Phylogenetic Analysis of Fish Digestive Lipases

**Figure 3:**
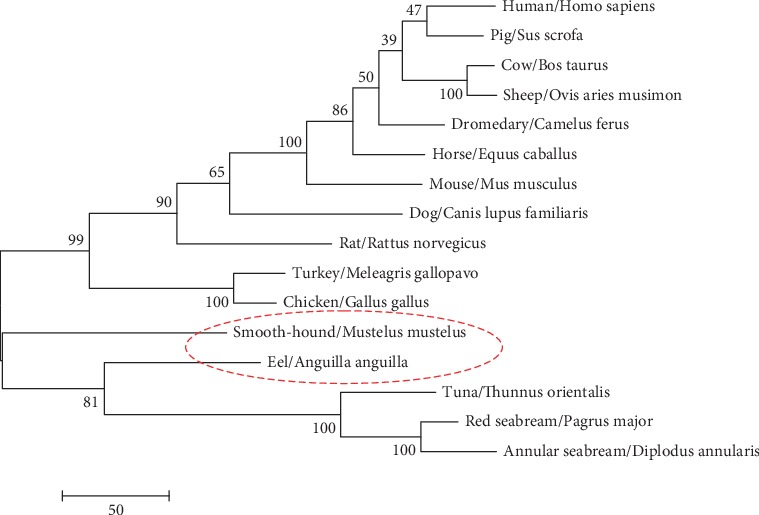
Maximum parsimony analysis of taxa. The evolutionary history was inferred using the maximum parsimony method. The percentage of replicate trees in which the associated taxa clustered together in the bootstrap test (100 replicates) is shown next to the branches [32]. The tree is drawn to scale with branch lengths calculated using the average pathway method [30] and being in the units of the number of changes over the whole sequence. The analysis involved 16 amino acid sequences. All positions containing gaps and missing data were eliminated. There were a total of 442 positions in the final dataset. Evolutionary analyses were conducted in MEGA6 [29].

We performed the phylogenetic analysis using 16 sequences: 11 classical pancreatic lipases (PL) and 5 fish digestive lipases (DL). A phylogenetic tree was constructed based on the alignment of the whole sequences (Figure 3). Based on the bootstrap values, all the obtained phylograms showed the same clustering of the sequences into two major clusters. According to the established phylogeny, the polypeptide sequences of smooth-hound and European eel digestive lipases were gathered together with those of tuna (*Thunnus orientalis*) and *Sparidae* lipases in one fish digestive lipase gene family (Figure 3). The phylogenetic tree also showed that lipases from annular and red seabreams and tuna took a similar evolutionary line separated in the early evolution from those of European Eel and smooth-hound which belong, respectively, to teleosts and the cartilaginous fish lipase subgroup. In 1999, Rasmussen and Amason have described the teleosts and the chondrichthyans as sister groups [37]. The phylogenetic tree showed that birds (chicken and turkey) are the closest to the cartilaginous fish; this behavior seems to be similar to those reported by Smichi et al. [24].

### 3.3. Modeling of SmDL and EeDL

The 3-D structure of the active human pancreatic lipase form (PDB code 1LPA) was utilized as a template to the build model for the open form of SmDL using the automated structure-modeling SWISS-MODEL workspace [34]. The generated 3-D models were then optimized by energy minimization. The Ramachandran plot statistics of the SmDL was carried out by the PROCHECK program and revealed that 97% of the residues were either in the most-favoured or in the additional authorized regions. A similar result (97.1%) was shown for the HPL structure. The fish structural model is well superposed to the HPL structure (Figure 1). The root-mean-squared deviations (RMSD) using alpha carbons were only 1.2 Å (419 atoms implicated) between the open forms of HPL and the fish digestive lipase. Like the HPL, the model of SmDL (open form) showed the presence of two domains: the catalytic N-terminal domain (residues 1 to 332) which is folded into a central parallel *β*-sheet consisting of 11 strands bounded by alpha helices on both sides and the *β*-sandwich C-terminal domain characterized by two antiparallel *β*-sheets (Figure 1). The 3-D models of SmDL and EeDL show that Ser153, Asp176, and His263 residues are located in a catalytic triad-like configuration (Figure 1). Like the HPL structure [10], the model of both enzymes contains six disulphide bridges. All structural features required for HPL activity and substrate recognition such as lid domain and *β*5 and *β*9 regions are also conserved in SmDL and EeDL structures (Figure 1). The fact that all the classical structural features are conserved in the 3-D models of SmDL and EeDL altogether suggest that these enzymes may share the same docket and the catalytic mechanism toward lipids. Thus, we have tried to bring more information on the digestive process of the SmDL.

### 3.4. SmDL Is a Serine Enzyme

**Figure 4:**
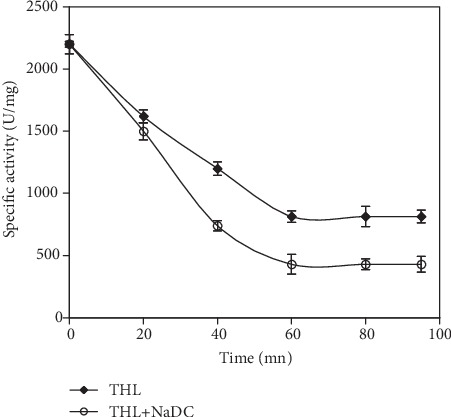
Inhibition of SmDL by THL in the absence and the presence of 4 mM NaDC. SmDL was incubated at 25°C with THL (molar ratio THL/SmDL = 100). SmDL activity was measured at pH 9 and 40°C using TC_4_ as a substrate.

The 3-D model of SmDL shows also that the amino acid key which is involved in the nucleophilic mechanism is Ser153 (Figure 4) which suggests that SmDL may be a serine enzyme. In order to verify if SmDL is a serine enzyme, we have used a serine inhibitor, THL, which acts as a potent inhibitor of gastric and pancreatic lipases, reacting with these enzymes' catalytic serine [38]. We studied the effect of THL on the SmDL activity [4]. The lipase lost 63% of its initial activity when incubated during 60 min with THL at a molar excess of 100. As described previously [39], NaDC addition (final concentration 4 mM) in the incubation medium accelerates significantly the inhibitory effect of THL. The mixed NaDC-THL micelles seem to be more appropriate than the THL alone for SmDL inhibition (Figure 4). We can conclude that SmDL is a serine enzyme, as all known lipases characterized from several sources [40, 41].

### 3.5. SmDL-Colipase Complex

#### 3.5.1. Biochemical Approach

**Figure 5:**
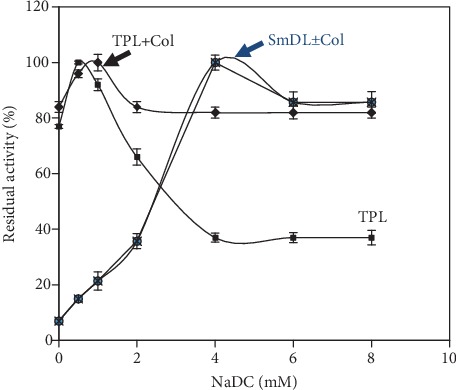
Effect of increasing the concentration of bile salts: NaDC. Lipolytic activity of SmDL and TPL was measured at pH 9 and 40°C using olive oil emulsion as a substrate in the presence and in the absence of a molar excess of colipase.

It is well established that all amphiphiles are strong inhibitors of pancreatic lipases [42]. Unlike classical lipases (HPL and TPL), the SmDL retained its maximal activity even at high bile salt concentrations reaching 8 mM [27]. Under these conditions, NaDC acts as an inhibitor of TPL activity when TC_4_ or olive oil emulsion is used as the substrate, but this inhibition is reversed by the addition of colipase. Thus, to check the presence of the classical colipase in the common smooth-hound pancreas, the homogenate was treated at pH 2 during 30 min, then at 60°C to retain only colipase activity. This colipase preparation succeeded to reactivate the classical TPL inhibited by bile salts (Figure 5). These results suggest that the common smooth-hound pancreas contains the classically known colipase. Considered as the most primitive living jawed vertebrates, cartilaginous fish, represented by sharks, might be considered as the oldest vertebrates having a complex digestive system the same as that of mammals. Surprisingly, the SmDL was not activated either by its own pH and/or temperature-treated homogenate (own colipase preparation) or by the turkey pancreatic colipase (Figure 5). However, in the same conditions, the TPL was activated. This suggests that the SmDL does not require interactions with colipase to be fully active. Similar results were reported for some bony fish digestive systems like grey mullet and *Sparidae* [43]. However, this behavior differs from that of mammal [16], avian [8], and stingray pancreatic [36] lipases, which are strongly inhibited by bile salts and reactivated by pancreatic colipase. As far as colipase activity was detected in the common smooth-hound pancreas, this may suggest that it produces another classical lipase, apart from SmDL, which is inactivated by bile salts and reactivated by colipase.

#### 3.5.2. Structural Approach: Colipase Potential Binding Sites

**Figure 6:**
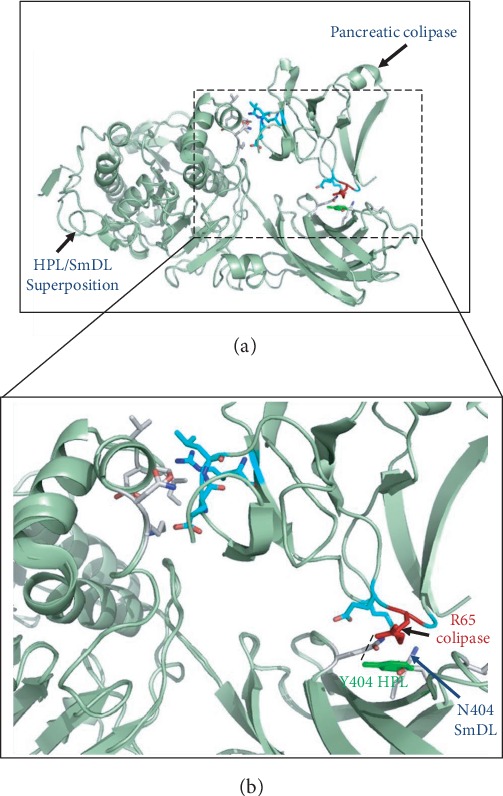
Lipase-colipase interaction. (a) Ribbon representation of SmDL and HPL interaction with colipase. The HPL structure is in complex with colipase (PDB code 1LPB). HPL and SmDL structures are well superposed. Residues involved in the lipase-colipase interaction are shown as sticks. (b) Zoom in from (a) showing the interaction interface between superimposed lipases (HPL and SmDL) and colipase. Residues conserved between the two lipases are shown only once. Residue Y404 in HPL and the corresponding residue in SmDL (N404) are both shown, and each of them is in interaction with colipase residue R65, shown in red. The hydrogen bond between R65 of the colipase and Y404 of HPL is shown as a dotted line.

Bile salts (NaDC) are physiological inhibitors of pancreatic lipases due to enzyme desorption. In the classical system, the colipase allows the lipase reactivation by anchoring the enzyme to the bile salt/lipid interface [44]. The colipase protein was characterized by an amphipathic overall that contains hydrophobic residues which are located at the tips of the fingers and interact with the lipidic substrate and hydrophilic residues that bind to the C-terminal domain of the enzyme [11]. The colipase was found to interact also with the open lid of pancreatic lipases [45]. It is well established that all lipase residues involved in colipase interaction are well conserved throughout the pancreatic lipase subfamily.  Table 2 shows that residues Asn241, Asp248, and Lys400 involved in HPL binding to colipase are conserved in the smooth-hound digestive lipase. Nevertheless, Ile402 which belongs to the C-terminal domain of HPL and interacts with Arg65 of colipase is substituted by Leu400 in the SmDL. Residues Asn241, Ser244, and Val247 of the lid are involved in interaction with Arg38, Leu16, and Glu15 of the colipase [45]. Val247 is changed to Ile247 in the SmDL. Interestingly, Tyr404 known to be a key residue for HPL binding to colipase [12] is replaced by Asn in the smooth-hound lipase (Table 2 and Figure 6). This Tyr404/Asn substitution was previously observed in the annular and red seabream digestive lipases (AsDL and RsDL) which are independent of the classical bile salt-colipase system [24]. In fact, HPL Tyr404 is stabilized through hydrophobic contacts with the bulky chain of Arg65 and also hydrogen bonding (by its OH group) to the Arg65 NH_2_ group (Figure 6). Tyr404/Asn substitution might disrupt the Tyr-Arg interaction and lead to uncomfortable contacts between the colipase arginine bulky chain and the polar lipase Asn groups which prevent the colipase binding to the SmDL. This might explain why SmDL is not reactivated by colipase. The Tyr404 of HPL and SmDL was replaced by Asn406 in the HPLRP2 (Table 2). As described by De Caro et al., this substitution might be responsible for weaker HPLRP2-colipase interactions, since HPLRP2 is inhibited by NaDC and partially reactivated by the pancreatic colipase [6, 46]. Furthermore, Xiao et al. [47] have shown that, unlike HPLRP2, porcine PLRP2 activity increases with increasing colipase concentration due to the conservation of the lid and C-terminal residues in the porcine PLRP2 sequence (including Tyr404) which is involved in the interactions of classical HPL with colipase. Altogether, these observations might give more insights on the absence of the classical system in the smooth-hound fish when assessing SmDL activity (Figure 6). The comparative studies of the lipolytic systems can be of great importance to understand the evolutionary relationship between animals, such as bony fish and cartilaginous fish. It has been shown that lower animals such as scorpions [18] and crabs [19] possess a combined hepatopancreas, whereas higher animals have well-differentiated separate livers and pancreases. This was thought to be the main evolutionary aspect of lipolytic systems. This work shows that another evolutionary fingerprint to consider is that of the colipase/bile salt/lipase system. Although they seem to be a homogenous digestive enzyme group, pancreatic lipases can be thereafter classified in two subgroups: colipase-dependent and colipase-independent enzymes. This works shows for the first time that a lipase from a cartilaginous fish (chondrichthyans) is interacting poorly with colipase. This was established for other fish belonging to bony fish. Based on this discovery, one can notice that the colipase/bile salt/pancreatic lipase system is evolving independently with the animal phylogeny. More studies on the composition and the evolution of bile salt composition and colipase sequence might give more insights on the discrepancies observed between animals and fish lipolytic systems. Moreover, this can open new perspectives in optimizing the application of lipolytic enzymes in bioconversion processes. A bile salt/colipase independent pancreatic lipase might be more easily applied as a biocatalyst as compared to a classical bile salt/colipase/lipase system.

## 4. Concluding Remarks

The genes encoding SmDL and EeDL were isolated for the first time from smooth-hound and European eel, respectively. The nucleotide BLAST analysis for SmDL indicated that it shared a high identity (77%–87%) with pancreatic lipase genes from some cartilaginous fish. In accordance with the established phylogeny, sequences of SmDL and EeDL genes were gathered together with those of tuna and *Sparidae* lipases in one fish digestive lipase cluster. The predicted 3-D structure of SmDL indicated the conservation of the signature features, such as the oxyanion hole, the lid, and the catalytic triad, shared among mammal pancreatic lipases. The substitutions of SmDL residues involved in the binding with colipase may destabilize the lipase-colipase complex in the open conformation. These contradictions may elucidate the fact that the classical pancreatic colipase is not efficient at activating the isolated lipase from the smooth-hound.

## Figures and Tables

**Table 1:** Primers used to amplify genes encoding for SmDL and EeDL.

Primers	Sequences 5′-3′
pSmDL-F	GCC GAA ATC TGC TAT AGC AG
pSmDL-R	TTA GCA AGG CAA AAC AGT TTG
pEeDL-F	GCC GAG GTG TGC TAT GAA AAC
pEeDL-R	TCA GCA CGG AGG AAC AGT CTG

**Table 2:** Main residues situated in the flap and the C-terminal domain involved in the binding of HPL to the porcine colipase. Corresponding residues in SmDL, PPLRP2, and HPLRP2, in porcine and cartilaginous fish colipase, are reported.

HPL	SmDL	AsDL	HPLRP2	Porcine colipase	Fish colipase
Asn241	Asn241	Asn238	Asn242	Glu15	Glu15
Ser244	Ser244	Gly240	Ser245	Leu16	Leu16
Val247	Ile247	Ser243	Thr248	Arg38	Arg38
Asp248	Asp248	Asp244	Asp248	Arg38	Arg38
Gln369	**Gln369**	**Glu367**	Gln371	**Glu64**	**Glu64**
Lys400	Lys400	Lys398	Lys403	Glu45	Glu45
Ile402	**Leu402**	Arg400	Leu404	Arg65	Arg65
Tyr404	**Asn404**	**Asn402**	**Asn406**	**Arg65**	**Arg65**

## Data Availability

The data used to support the findings of this study are available from the corresponding author upon request.

## Abbreviations

SmDL: Smooth-hound digestive lipase
EeDL: European eel digestive lipase
HPL: Human pancreatic lipase
HPLRP1: Human pancreatic lipase-related protein 1
HPLRP2: Human pancreatic lipase-related protein 2
AsDL: Annular seabream digestive lipase
RsDL: Red seabream lipase
PPLRP2: Porcine pancreatic lipase-related protein 2
TPL: Turkey pancreatic lipase
RT-PCR: Reverse transcription-polymerase chain reaction
THL: Tetrahydrolipstatin
NaDC: Sodium deoxycholate.

## Supplementary Materials

Supplementary Materials Figure S1: (a) agarose gel electrophoresis (1.5%) of the RNA extraction. Lane 1 contains 1 kb DNA molecular weight markers; lanes 2 and 3 contain the RNA of smooth-hound and European eel pancreases. (b) Agarose gel electrophoresis (1%) of the PCR products of fish digestive lipases. Lane 1 contains 1 kb DNA molecular weight markers; lanes 2 and 3 contain the PCR products of smooth-hound and European eel digestive lipases, respectively. Figure S2: nucleotide sequences of the gene encoding the mature SmDL (a) and EeDL (b) and the deduced amino acid sequences of correspondent lipases.
