# Supplementary material for: Dissecting the Interaction Deficiency of a Cartilaginous Fish Digestive Lipase with Pancreatic Colipase: Biochemical and Structural Insights
Source: Biomed Res Int. 2020 Mar 13;2020:3064290. doi: 10.1155/2020/3064290 (PMC7094207; doi:10.1155/2020/3064290)
Supplement: Supplementary Materials — Figure S1: (a) agarose gel electrophoresis (1.5%) of the RNA extraction. Lane 1 contains 1 kb DNA molecular weight markers; lanes 2 and 3 contain the RNA of smooth-hound and European eel pancreases. (b) Agarose gel electrophoresis (1%) of the PCR products of fish digestive lipases. Lane 1 contains 1 kb DNA molecular weight markers; lanes 2 and 3 contain the PCR products of smooth-hound and European eel digestive lipases, respectively. Figure S2: nucleotide sequences of the gene encoding the mature SmDL (a) and EeDL (b) and the deduced amino acid sequences of correspondent lipases. [file 3064290.f1.docx]

**
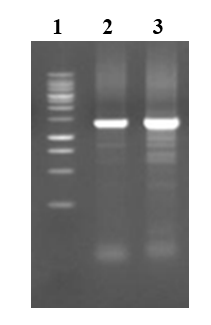
Figure S1**

**
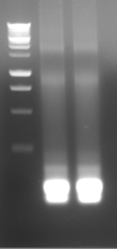
**

**1350 bp**

**B**

**1 2 3**

**A**

**1 2 3**

**ARNr 18S**

**ARNr 28S**

**Fig. S1. (A),** Agarose gel electrophoresis (1.5%) of the RNA extraction. Lane 1 contains 1kb DNA molecular weight markers; lanes 2 and 3 contain the RNA of smooth-hound and European eel pancreases. (**B)** Agarose gel electrophoresis (1%) of the PCR products of fish digestive lipases*.* Lane 1 contains 1kb DNA molecular weight markers; lanes 2 and 3 contain the PCR products of smooth-hound and European eel digestive lipases, respectively.

**Figure S2A**

gccgaaatctgctatagcagattaggctgctttacggatgatgttccatggggtgggaca

A E I C Y S R L G C F T D D V P W G G T

ccacaaagaccagttaaaaaattaccctggtccccagaggatattaacacacgtatgttg

P Q R P V K K L P W S P E D I N T R M L

ctttggacaagacgaaatacagcaacttttcaggaaattaccgacatgactccttcaaca

L W T R R N T A T F Q E I T D M T P S T

atacaaaactctaattttgaccggaccaaaaaaacccgcttcatagttcatggatatgta

I Q N S N F D R T K K T R F I V H G Y V

gacaagggagaagaatcgtggctctctgatatgtgcaaggcaatgtttcaagtggaagat

D K G E E S W L S D M C K A M F Q V E D

gtgaattgtatttgtgttgattgggtccgtggttcaagaaccctatacgatcaggctgct

V N C I C V D W V R G S R T L Y D Q A A

aacaacatccgggtggttggtgcagagatagcatatcttattgatgtgttagagaaaaat

N N I R V V G A E I A Y L I D V L E K N

tctaactactcacgttccgagacccacattattgggcacagtttgggcagccacgctgca

S N Y S R S E T H I I G H S L G S H A A

agtgaaactggaagacgccttccagggatcggaagaatcacaggtctagatccagccaaa

S E T G R R L P G I G R I T G L D P A K

cccttttttaagaacactcccattgaagttcgactagatacttcagatgcagtgtttgtt

P F F K N T P I E V R L D T S D A V F V

gacattatccacactaatgcagctccattgcttcctcaccttggttttggattactcgaa

D I I H T N A A P L L P H L G F G L L E

cccattggacatcttgacttctatccaaatggtggagaattaatgcctggatgtgacaaa

P I G H L D F Y P N G G E L M P G C D K

aacattgcttcaaccgtaattgatattaatggcatttgggaaggtactcacaattttgca

N I A S T V I D I N G I W E G T H N F A

gcatgcagccacttacgatcatataaatattacacggcgagcatcacaaccaaaactgga

A C S H L R S Y K Y Y T A S I T T K T G

ttcatgggtattccgtgtaagagctatgatgactttgaagcagggaattgtttcagctgc

F M G I P C K S Y D D F E A G N C F S C

cctccagaaggctgccccacaatgggtcactttgctaacacctaccctcttgggaatgtt

P P E G C P T M G H F A N T Y P L G N V cccactgaactcagtttttatttgaacacgggtgctgctccgtcttttgcacgctggcga

P T E L S F Y L N T G A A P S F A R W R

tacaaggttactgttaaaattacctgtacacggagtatcagaggattcttcaatattgcc

Y K V T V K I T C T R S I R G F F N I A

ctgtatggttccaatgtcaatacccggcaatatcaagttgcaaaagcattgttggcatct

L Y G S N V N T R Q Y Q V A K A L L A S

ggaaaaacattcactgctgagatcgacgtggaaaaagatttagcagaggttactaaagtc

G K T F T A E I D V E K D L A E V T K V

aaatttctctggaataatgttatgccaaatcttctcaggccaaaagttggagcagaaact

K F L W N N V M P N L L R P K V G A E T

gtaaccctattaagagtatatgaccagcaaacctttaggttttgtggaactgggcgtgtt

V T L L R V Y D Q Q T F R F C G T G R V

ggcgaagatgttctgcaaactgttttgccttgctaa

G E D V L Q T V L P C -

**Figure S2B.**

gccgaggtgtgctatgaaaacctgggctgtttcacggacgacgttccctgggcgggcacc

A E V C Y E N L G C F T D D V P W A G T

accgagcgaccaatcgccaagctcccctggagcccagagaagataggcacccgcttcatg

T E R P I A K L P W S P E K I G T R F M

ctcttcacccggcagaaccccaacaaccaccaggagatcacaacaaaagaggacatcctt

L F T R Q N P N N H Q E I T T K E D I L

ctggcatcgaattacgacgggaccaggaagacgcgcttcattacccatggctacgtcgac

L A S N Y D G T R K T R F I T H G Y V D

aaaggggatgaaaattggctgattgacatgtgcaagctgatgctccaagtggaggacatc

K G D E N W L I D M C K L M L Q V E D I

aactgcatctgcgtggactggaagaagggaggccggaccctgtacacgcagagcgccagc

N C I C V D W K K G G R T L Y T Q S A S

aacatccgcgtcatcggggcccagacggcctacatgatccagctgttccagacgctgtac

N I R V I G A Q T A Y M I Q L F Q T L Y

cagcagaggcccgagtcggtccacatcatcgggcacagcctgggggcgcactgtgcaggc

Q Q R P E S V H I I G H S L G A H C A G

gaggccgggcgcaggaccccaaacctgggccgcatcaccggtctggatcctgccgagccg

E A G R R T P N L G R I T G L D P A E P

tacttccagggctgccccagcctggtgcgcctggaccccagcgacgccaagttcgtggac

Y F Q G C P S L V R L D P S D A K F V D

gtcattcacacagacgcgaaacccatgattccctatcttgggatgggaatggctcaggct

V I H T D A K P M I P Y L G M G M A Q A

gtcgaccatcttgacttctaccctaacggaggggagcacatgcctggatgcgacaagaac

V D H L D F Y P N G G E H M P G C D K N

ctcatctctcagattgtggacatcgacggcatttgggaaggcacccgtgactttgtggcc

L I S Q I V D I D G I W E G T R D F V A

tgcaaccacctgaggtcctacaagtactacagcgacagcatcctgaacccagagggattc

C N H L R S Y K Y Y S D S I L N P E G F

actgggtacccctgctccgacggaggcgtcttcgagtccggtcgctgtttcccctgcggg

T G Y P C S D G G V F E S G R C F P C G

gacggggcgtgtcccttcatgggtcaccatgccgacaagttccgccggccgaacggggcg

D G A C P F M G H H A D K F R R P N G A

gagaagatgaagttctacctgaacaccgccgacgccaagcctttcggtcggtttcgctac

E K M K F Y L N T A D A K P F G R F R Y

aaagtgacggtcacaatcaggggtaaccgagcgcttctcctgactgggacaatgagcgtg

K V T V T I R G N R A L L L T G T M S V

gccatctacgggacccaaggcaacaccagacaatatcagattagaaagggtctccttaag

A I Y G T Q G N T R Q Y Q I R K G L L K

cccgggaacacgtacgaagcctacatcgacaccgagactgatgcaggcgaagtgaccaaa

P G N T Y E A Y I D T E T D A G E V T K

atgaagttcatctgggacaacagcgtcatcaaccccttgttccccaagcttggcgctgag

M K F I W D N S V I N P L F P K L G A E

aagatcgtcctgcaacggggaaaggacaggcgcgttttctcattctgtagctccgagacc

K I V L Q R G K D R R V F S F C S S E T

gagcgtgaggaagtgctgcagactgttcctccgtgctga

E R E E V L Q T V P P C -

**Fig. S2.** Nucleotide sequences of the gene encoding the mature SmDL (**A**) and EeDL (**B**) and the deduced amino acid sequences of correspondent lipases.
